# Retraction: Correlation of FIRE-MADE (Frailty Index in Rural Elderly - Mental Status, Activities of Daily Living, Depression, and Events) With Sarcopenia in Elderly Population of Central Rural India

**DOI:** 10.7759/cureus.r212

**Published:** 2025-12-18

**Authors:** Khalid I Khan, Shilpa A Gaidhane, Sunil Kumar, Sourya Acharya

**Affiliations:** 1 Medicine, Jawaharlal Nehru Medical College, Datta Meghe Institute of Medical Sciences, Wardha, IND

This article has been retracted by the Editors-in-Chief due to a copyright violation by the authors concerning the unauthorized use of the Mini-Mental State Examination (MMSE). As a result, this article has been retracted and the article contents have been removed.

